# Prevalence of e-cigarette users in the Medina region of Saudi Arabia

**DOI:** 10.18332/tid/209586

**Published:** 2025-09-29

**Authors:** Noof Aloufi, Renad M. Alhamawi, Shahad N. Alalwani, Wateen K. Alrefaei, Hana A. Aljohani, Mayan M. Ali, Fahad H. Alahmadi

**Affiliations:** 1 Department of Clinical Laboratory Sciences, College of Applied Medical Sciences, Taibah University, Madinah, Saudi Arabia; 2 Department of Respiratory Therapy, College of Medical Rehabilitation Sciences, Taibah University, Madinah, Saudi Arabia

**Keywords:** vaping, e-cigarettes, young adult, fruit flavor

## Abstract

**INTRODUCTION:**

Electronic cigarettes are battery-operated devices that allow users to ‘vape’ flavored solutions including nicotine. The prevalence of users of e-cigarettes with different flavors, is not known in the Medina region in Saudi Arabia. Thus, the study aimed to assess the prevalence and characteristics of e-cigarette smokers in Medina region. Additionally, this study aimed to determine the popular flavors and the most common reasons for smoking e-cigarettes among young adults.

**METHODS:**

This cross-sectional study was conducted between 1 February and 19 March 2024 using an online questionnaire via convenience sampling. The total number of participants was 388, including males and females aged ≥18 years.

**RESULTS:**

The study findings showed that 78.2 % of the e-cigarette smokers were young adults, while 58.4 % of the participants aged 18–29 years preferred fruit flavors. Additionally, the majority of young adults used e-cigarettes for reducing stress and enjoyment, corresponding to 40.6 % and 31.7 % of participants, respectively.

**CONCLUSIONS:**

Our study is useful for identifying the preferences towards e-cigarette usage within a population and monitoring emerging trends, particularly among young adults. Researching regional preferences for e-cigarette flavors might help to direct future studies into the health effects of various flavorings.

## INTRODUCTION

The use of electronic cigarettes, referred to as vape devices or electronic nicotine delivery systems (ENDS), has increased significantly worldwide^1,2^. The market is also rapidly targeting young people, with a wide range of products and attractive flavors^3,4^. E-cigarettes are defined as battery-operated items that deliver nicotine in the form of an aerosol. Using e-cigarettes differs from typical cigarette smoking, which includes burning and inhaling tobacco smoke^1^. E-cigarettes consist of a cartridge, an atomizer, and a mouthpiece. The cartridge is a reservoir that is filled with a liquid^5^. The primary ingredients of e-liquid are vegetable glycerol and propylene glycol, nicotine, and often flavors^5^. The chemicals found in the aerosol generated from e-cigarette solutions are formaldehyde, acetaldehyde, acrolein, and benzaldehyde^6^. An approximate 7700 e-liquids are available, each with a different concentration of nicotine and/or flavoring, from 466 different companies^7^. The flavors of e-liquids are generally categorized into fruit, candy, tobacco, menthol or mint, and coffee, consumed most commonly by e-cigarette users^8^.

Numerous studies have demonstrated the association between respiratory disorders and e-cigarettes^1,9,10^. It has been suggested that using e-cigarettes aids in the emergence or persistence of respiratory symptoms or the change from acute symptomatology to a chronic illness^1,11^. In Saudi Arabia, Taif University found that e-cigarette consumption was higher among males aged 18–24 years^12^. However, the extent of the harmful effects of e-cigarettes with different flavors in Saudi Arabia is not known. The prevalence of vapers using different popular flavors among young adults in the Medina region is not known. With the increase in popularity and marketing of e-cigarette usage, this study hypothesized that e-cigarette usage with different flavors, such as fruits, has increased among young adults. Therefore, in our study, we aim to assess the prevalence of smokers of e-cigarettes with different flavors, in the population of the Medina region across a wide age range.

## METHODS

### Study design and population

This cross-sectional study was conducted between 1 February and 19 March 2024 via online survey, Google Forms. This study included a convenience sample of male and female adults, aged ≥18 years in the Medina region, who were categorized as never smokers, tobacco cigarette smokers, e-cigarette smokers, or hookah smokers. An institutional research ethics board approval was obtained from Taibah University before starting the study (2024/186/105 MLT). All participants (n=392) signed a consent form to participate in the study. The identity of the participants was kept confidential. We excluded subjects who were aged <18 years (4 participants); thus, the final included participants were 388.

### Data collection

The recruitment was done by sending advertisements to participate in the study through social media such as WhatsApp, X, public emails, etc. Data were collected through an online, detailed self-reporting questionnaire, which was sent via social media. The questionnaire involved different categories relating to sociodemographic data (age, gender, nationality, education level, and marital status) and general health questions (diseases, allergies, surgeries, and medical drugs). The questionnaire also involved questions related to smoking status as well as e-cigarette-related questions, including type of e-cigarette, behaviors, flavors, age when starting to vape, and reasons for consuming e-cigarettes. The smoking status was categorized based on responses from participants who had smoked tobacco, e-cigarette, or hookah during the past 30 days. The questionnaire questions were adopted and modified from the Canadian Tobacco and Nicotine Survey^13^. The questionnaires are provided in the Supplementary file. The height and weight were also asked to assess the body mass index (BMI).

### Statistical analysis

All statistical analyses and graphical representations were performed using GraphPad Prism version 9 and SPSS version 20 (SPSS, Inc., Chicago, IL, USA). Descriptive statistics are shown as frequencies and percentages for categorical data, and means ± standard deviation (SD) for continuous variables, unless otherwise indicated. Comparisons of variables between never smokers and e-cigarette smokers were performed using the Fisher’s exact test for categorical variables. In all cases, a p<0.05 was considered statistically significant.

## RESULTS

### The prevalence of e-cigarette consumers among the population in the Medina region

A complete survey was obtained of 388 participants. We assessed the prevalence of e-cigarette users among our study population, and we found that 214 (55.2%) were never smokers, 101 (26%) were e-cigarette smokers, 48 (12.4%) were tobacco cigarette smokers, 22 (5.7%) were hookah smokers, and 3 (0.8%) were tobaccoless products users (data not shown).

Table 1 demonstrates the demographic characteristics and exposure types in the study population. In all, 34.1% of males and 65.9% of females had never smoked, 67.3% of males and 32.7% of females had smoked e-cigarettes, 75% of males and 25% of females had smoked tobacco, and 45.45% of males and 54.55% of females had used hookahs. In terms of nationality, 92.5% of Saudis and 7.5% of non-Saudis had never smoked, 96% of Saudis and 4% of non-Saudis had smoked e-cigarettes, 98% of Saudis and 2% of non-Saudis smoked tobacco, and 100% of Saudis smoked hookah.

**Table 1:** Demographic characteristics, by smoking status, of participants in an online survey, Medina region, Saudi Arabia, 1 February and 19 March 2024 (N=388)

*Characteristics*	*Categories*	*Never smoker* *(N=214)* *n (%)*	*E-cigarette smoker* *(N=101)* *n (%)*	*Tobacco smoker* *(N=48)* *n (%)*	*Hookah smoker* *(N=22)* *n (%)*
**Gender**	Male	73 (34.1)	68 (67.3)	36 (75)	10 (45.5)
	Female	141 (65.9)	33 (32.7)	12 (25)	12 (54.5)
**Nationality**	Saudi	198 (92.5)	97 (96)	47 (98)	22 (100)
	Non-Saudi	16 (7.5)	4 (4)	1 (2)	0
**Age** (years)	18–29	131 (61.2)	79 (78.2)	24 (50)	9 (41)
	30–39	31 (14.5)	15 (14.9)	14 (29.2)	7 (31.8)
	40–49	27 (12.6)	2 (2)	4 (8.3)	3 (13.6)
	50–59	19 (8.9)	5 (5)	5 (10.4)	3 (13.6)
	≥60	6 (2.8)	0	1 (2.1)	0
**Education level**	≤ High school	78 (34.4)	31 (30.7)	15 (31.3)	3 (13.6)
	Diploma/ University/Postgraduate	136 (63.6)	70 (69.3)	33 (68.8)	19 (86.4)
**Marital status**	Single	131 (61.2)	82 (81.2)	30 (62.5)	10 (45.5)
	Married	83 (38.8)	19 (18.8)	18 (37.5)	12 (54.5)
**BMI**, mean ± SD		24.6 ± 5.7	24.7 ± 5.6	26.7 ± 5.8	25.7 ± 5.4

BMI: body mass index (kg/m^2^).

Additionally, the mean ± SD of self-reported body mass index (BMI, kg/m^2^) in non-smokers was 24.6 ± 5.7, whereas e-cigarette smokers had 24.7 ± 5.6, both of which are considered healthy weights. Tobacco smokers had a BMI of 26.7 ± 5.8, whereas hookah smokers had a BMI of 25.7 ± 5.4 (Table 1).

Table 2 shows the demographic characteristics in the study population stratified by smoking status. Compared with never smokers, male e-cigarette smokers were similar. However, female never smokers were more compared to female e-cigarette smokers.

**Table 2:** Characteristics of never smokers and e-cigarette smokers, of participants in an online survey, Medina region, Saudi Arabia, 1 February and 19 March 2024 (N=315)

*Characteristics*	*Categories*	*Never smokers* *(N=214)* *n (%)*	*E-cigarette smokers* *(N=101)* *n (%)*	*p*
**Gender**	Male	73 (51.8)	68 (48.2)	0.0001*
	Female	141 (81.0)	33 (19.0)	
**Nationality**	Saudi	198 (67.1)	97 (32.9)	0.323
	Non-Saudi	16 (80.0)	4 (20.0)	
**Age** (years)	18–29	131 (62.4)	79 (37.6)	0.002*
	30–39	31 (67.4)	15 (32.6)	
	40–49	27 (93.1)	2 (6.9)	
	50–59	19 (79.2)	5 (20.8)	
	≥60	6 (100)	0 (0.0)	
**Education level**	≤ High school	78 (71.6)	31 (28.4)	0.375
	Diploma/ University/postgraduate	136 (66.0)	70 (34.0)	
**Marital status**	Single	131 (61.5)	82 (38.5)	0.0001*
	Married	83 (81.4)	19 (18.6)	

* Significant at 95% confidence level. Fisher’s exact test was performed to obtain the data provided in the Table.

Additionally, we identified the age at which each participant started using e-cigarette and found that 35.6% of males and 12.9% of females started vaping when they were aged <20 years; 22.8% of males and 14.9% of females started vaping aged 20–29 years, as well as 4% of males and 5% of females started vaping when they were aged 30–39 years. In the age groups 40–49 years and 50–59 years, 3% and 2% of males started vaping, respectively (data not shown).

### Patterns of e-cigarette use

One of the questions in our survey was about the patterns of e-cigarette usage. Table 3 shows that most e-cigarette users used them daily. In detail, 66.3% of e-cigarette participants who use e-cigarettes every day were young adults (aged 18–29 years). Regarding the next question, 54.5% of e-cigarette users were young adults who picked up their e-cigarette to vape at least 6 times. The participants were also asked about how many puffs they usually inhaled during their vaping. We found that 34.7% of e-cigarette users were young adults who took at least 6 puffs for each usage.

**Table 3:** Behavior of e-cigarette smokers, by age (years) group, of participants in an online survey, Medina region, Saudi Arabia, 1 February and 19 March 2024 (N=101)

*Question*	*Response*	*18–29* *n (%)*	*30–39* *n (%)*	*40–49* *n (%)*	*50–59* *n (%)*
**During the past 30 days, how often did you vape?**	Daily	67 (66.3)	15 (14.9)	1 (1)	4 (4)
	Less than daily, but at least once a week	7 (6.9)	0	1 (1)	1 (1)
	Less than once a week, but at least once a month	5 (5)	0	0	0
**How many times do you usually pick up or take your e-cigarette to vape in a day?**	1 time	8 (7.9)	0	1 (1)	0
	2 times	6 (5.9)	0	0	0
	3–5 times	10 (9.9)	3 (3)	0	1 (1)
	≥6 times	55 (54.5)	12 (11.9)	1 (1)	4 (4)
**Each time you use your e-cigarette to vape, how many puffs do you usually take before putting it away?**	1	10 (9.9)	1 (1)	0	1 (1)
	2	15 (14.9)	2 (2)	0	0
	3–5	19 (18.8)	6 (5.9)	1(1)	2 (2)
	≥6	35 (34.7)	6 (5.9)	1(1)	2 (2)

### Fruit flavor is the most popular flavor among young adults

**Figure 1:**
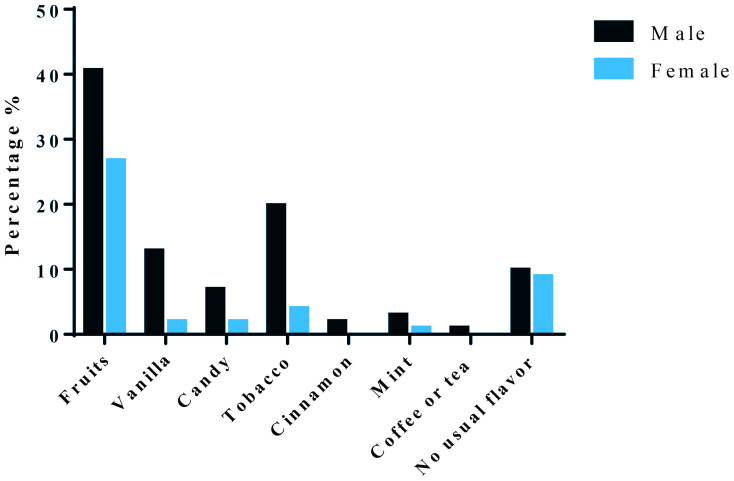
Percentage of different flavors that are popular for males and females (N=101)

Our questionnaire also included a question about which flavor is popular. It was noted that 40.6% of male versus 26.7% of female vapers preferred fruit flavors. Furthermore, 19.8% of males vs 3.9% of females used tobacco flavors as well as 9.8% of males and 8.8% of females did not have a usual flavor. Candy flavor was also used by males with a percentage of 6.9% and by females with a percentage of 2%. Finally, the least preferred flavors were mint, coffee or tea and cinnamon (Figure 1).

**Figure 2:**
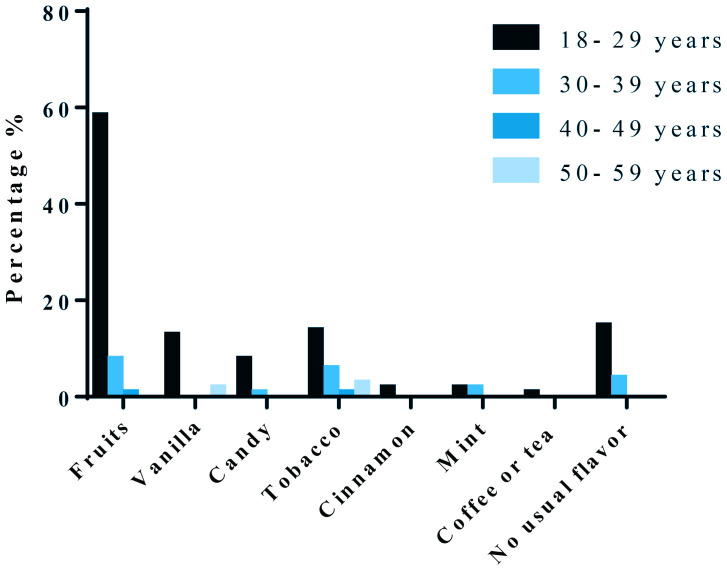
Percentage of different ages and their favorite flavors (fruit, vanilla, candy, tobacco, cinnamon, mint, coffee or tea, no usual flavor) among the study population (N=101)

We also evaluated the different popular flavors according to age. We discovered that 58.4% of the participants aged 18–29 years preferred fruits flavors, 12.9% preferred vanilla flavor, 0.9% preferred candy flavor, 13.9% preferred tobacco flavor and around 14% did not have usual flavors. The participants aged 30–39 years liked fruits and tobacco flavors with percentage of 8% and 6%, respectively. The age group 40–49 years preferred also fruits and tobacco flavors with percentage of around 1% for both flavors. Finally, the age group 50–59 years preferred also fruits and tobacco flavors with percentage of 2% and 3%, respectively (Figure 2).

### Enjoyment and reducing stress are the most common reasons of using e-cigarettes among young adults

In our survey, we asked the participants about their primary motives for vaping through a multiple-choice question, and the responses are categorized by age groups (Table 4). Among respondents, those aged 18–29 years, 40.6% and 31.7% cited reducing stress or calming themselves down and enjoyment, respectively, as their main motives for using e-cigarettes. In the group aged 30–39 years, 8.9% reported using e-cigarettes to quit smoking tobacco cigarettes as their main reason. Participants aged 50–59 years prefer using e-cigarettes because they taste better (3% of participants) and it is less harmful (2% of participants) compared to tobacco smoking (Table 4).

**Table 4:** The main reasons for using e-cigarettes, by age (years) group, of participants in an online survey, Medina region, Saudi Arabia, 1 February and 19 March 2024 (N=101)

*Reasons*	*18–29* *n (%)*	*30–39* *n (%)*	*40–49* *n (%)*	*50–59* *n (%)*
Curiosity, you just wanted to try it	20 (19.8)	0	0	0
Because you enjoy it	32 (31.7)	3 (3)	1 (1)	0
To reduce stress or calm you down	41 (40.6)	4 (3.9)	0	0
To quit smoking tobacco cigarettes	15 (14.9)	9 (8.9)	0	0
To reduce the use of smoking tobacco cigarettes	9 (8.9)	4 (4)	0	0
To use when you cannot or are not allowed to smoke cigarettes	6 (5.9)	3 (3)	0	1 (1)
To use with family members or friends	12 (11.9)	2 (2)	0	0
Because you think it’s less harmful	17 (16.8)	4 (4)	1 (1)	2 (2)
Because it tastes better than smoking tobacco cigarettes	20 (19.8)	4 (4)	0	3 (3)

Moreover, we have asked the participants about how many times they have stopped vaping for one day or longer during the past 12 months. Interestingly, 40.6% of e-cigarette users did not try to quit vaping at all (data not shown). We have also asked the vapers about their opinion regarding the harmful effects of e-cigarettes on a person’s health. We found that 26.7% of respondents thought that smoking e-cigarettes is harmful, the same as tobacco cigarettes, and 23.8% thought that e-cigarettes are more harmful than tobacco cigarettes. However, 40.6% thought that e-cigarettes are less harmful than tobacco cigarettes (data not shown).

## DISCUSSION

This cross-sectional study was conducted to demonstrate the prevalence of e-cigarette smoking in the Medina region. According to our findings, young adults were the majority of e-cigarette users with a preference for fruit flavors. Most of the young adults used e-cigarettes for enjoyment and to reduce stress.

Flavored e-cigarettes offer a wide range of flavors for vaping, such as fruit, sweet, and savory options^8^. These flavors enhance the enjoyment of vaping for young people. For instance, mango and mint were used most often among adolescents in the United States^14^. Previous studies conducted in Saudi Arabia did not discuss flavors^12,15-17^, while our present study thoroughly addressed the favored flavors among young adults in the Medina region. Our study revealed that fruits emerged as the predominant flavor category favored by young adults. A similar study in the United States found that young adults preferred fruit flavors, while older adults preferred tobacco flavors^18^. This discrepancy underscores targeted marketing strategies and evolving trends within the vaping industry. This suggests that different age groups have distinct preferences when it comes to e-cigarette flavors.

E-cigarette solutions with fruit and dessert flavors increased the rewarding and reinforcing value of vaping among young adults compared to unflavored ones, even with constant nicotine concentrations^19^. Further, young adults aged 18–24 years were more inclined to start vaping due to flavors, especially fruit flavors, compared to adults aged 35–44 years^20^. Users of flavored e-cigarettes also reported more satisfaction and self-perceived addiction compared to those using non-flavored e-cigarettes^20^. These findings highlight the potential impact of flavors in attracting young people to vape and the need for stricter regulations to protect their health, and reinforce the value of vaping for young adults.

Furthermore, our survey respondents were asked about the main reasons for their use of e-cigarettes, and the top two reasons were enjoyment and reducing stress among young adults. Adults were more likely to report using e-cigarettes for smoking cessation and because they thought it is more acceptable and less expensive than tobacco cigarettes, whereas adolescents and young adults were more likely to report using e-cigarettes out of curiosity and because it tasted good^21^. Youth e-cigarette users and youth social media users have all gained significant attention in the public health arena in recent years^22^. Additionally, adolescents’ utilizing of social media is linked to a higher chance of using e-cigarettes^23^. Indeed, social media has been involved in the rise of e-cigarette popularity by drawing its attractive characteristics and providing a platform for youth to express and communicate about vaping^24^. Promotion and usage of e-cigarettes are common on many social media sites, including YouTube and Instagram, where videos may be viewed by millions of people, some of whom may be minors^25,26^. These findings suggest that social media is very important to the e-cigarette business.

A prior study has demonstrated a correlation between psychological distress and e-cigarette use^27^. Thus, research on the significant impacts of short- and long-term e-cigarette users on human health would be beneficial. Because of the growth in the prevalence of e-cigarettes, it is vital to plan and execute various educational initiatives to raise knowledge of the consequences of e-cigarettes on health. According to the Pan American Health Organization (PAHO), eight nations have banned the sale of e-cigarettes, while the remaining 13 have partially or fully enacted one or more regulatory measures^28^.

### Limitations

There are some limitations to this study, primarily related to the sample size and recruitment strategy. First, our sample size was based on the available population and logistical feasibility. Second, the use of social media for participant recruitment may have contributed to selection bias, resulting in a disproportionate representation of younger individuals and a lower participation rate from those aged ≥40 years. Further, we did not perform formal reliability or internal consistency testing on the questionnaire prior to data collection. However, the questionnaire used in our study was adapted from a previously published study from Canada^13^.

## CONCLUSIONS

Our study indicates potential preferences of e-cigarette flavors in the Medina region. This is among the first studies in the region to focus specifically on flavor preferences among younger adult users, including frequency and reasons for usage stratified by age group. Our study is useful for identifying preferences for e-cigarette use within a population and monitoring emerging trends, particularly among young adults. Researching regional preferences for e-cigarette flavors might help to direct future studies into the health effects of various flavorings.

## Supplementary Material



## Data Availability

The data supporting this research are available from the authors on reasonable request.
