# Supplementary material for: Prevalence of e-cigarette users in the Medina region of Saudi Arabia
Source: Tob Induc Dis. 2025 Sep 29;23:10.18332/tid/209586. doi: 10.18332/tid/209586 (PMC12477758; doi:10.18332/tid/209586)
Supplement: Supplementary file 1 [file TID-23-145-s1.pdf]

Supplementary Materials and Methods:

The cross-sectional study was conducted between 1 February 2024 to 19 March 2024 via online survey, Google Forms. The questions of the questionnaire include the following:

1)- Demographic data:

Q1: What is your Sex?

1. Male
2. Female

Q2: How old are you?

Q3: What is your nationality?

1. Saudi
2. Non-Saudi

Q4: What is the highest certificate, diploma or degree that you have completed?

1. Less than high school
2. High school certificate
3. Diploma
4. Bachelor's degree
5. Master’s
6. PhD

Q5: What is your marital status?

1. Single
2. Married

Q6- Where do you live (Residency)?

Madinah, Makkah, Riyadh, Eastern Region, Najran, Tabuk, Northern Borders, Hail, Jouf, Asir, Qassim, Jazan, and Baha

2)- Smoking status:

Q1: During the past 30 days, did you use one of the following products?

1. E-liquid with nicotine
2. E-liquid with no nicotine
3. E-liquid with don’t know what contains
4. Tobacco cigarette
5. Hookah
6. Tobaccoless product
7. Others, please specify

3)- Vaping or E-cigarette usage related questions:

Q1: How old were you when you first tried vaping?

1. Less than 20 years
2. 20- 29
3. 30- 39
4. 40- 49
5. 50- 59

6. 60 years old or above

Q2: During the past 30 days, how often did you vape?

1. Daily
2. Less than daily, but at least once a week
3. Less than once a week, but at least once in the past month

Q3: How many times do you usually pick up or take out your e-cigarette to vape in a day?

1. 1 time
2. 2 times
3. 3 to 5 times
4. 6 or more times

Q4: Each time you use your e-cigarette to vape, how many puffs do you usually take before putting it away?

1. 1 puff
2. 2 puffs
3. 3 to 5 puffs
4. 6 or more puffs

Q5: Which flavor do you vape most often?

1. Tobacco
2. Watermelon
3. Berries
4. Candy
5. Cinnamon
6. Dessert
7. Mint or menthol
8. Coffee or tea
9. Flavourless
10. No usual flavour
11. Other, please specify

Q6: If you are an e-cigarette smoker, do you smoke tobacco cigarettes at the same time?

1. Yes.
2. No.

Q7: If you are an e-cigarette smoker, do you use tobaccoless products at the same time?

1. Yes.
2. No.

Q8: What is your main reason for vaping?

1. By curiosity, you just wanted to try it
2. Because you enjoy it
3. To reduce stress or calm you down
4. To quit smoking tobacco cigarettes
5. To reduce the use of smoking tobacco cigarettes

6. To use when you cannot or are not allowed to smoke cigarettes
7. To use with family members or friends
8. Because you think it is less harmful
9. Because it tastes better
10. Other, please specify

Q9: During the past 12 months, how many times have you stopped vaping for one day or longer because you were trying to quit vaping?

1. 3 times
2. 5 times
3. 6 or more times
4. Not at all

Q10: In your opinion, compared with cigarettes, how harmful to a person's health are e-cigarettes?

1. Much less harmful than tobacco cigarettes
2. Somewhat less harmful than tobacco cigarettes
3. About the same as tobacco cigarettes
4. Somewhat more harmful than tobacco cigarettes
5. Much more harmful than tobacco cigarettes
6. Do not know

4)- General Health:

Q1: Do you suffer from any chronic diseases/ conditions?

Please specify

Q2: Do you use any medications?

Please specify

Q3: Have you ever had any surgical procedures or interventions?

1. Yes, please specify
2. No

5)- Height and Weight:

Q1: Please provide your height.

Q2: Please provide your weight.

© 2025 Aloufi N. et al.
